# Suprachoroidal space-inducing hydrogel-forming microneedles (SI-HFMN): An innovative platform for drug delivery to the posterior segment of the eye

**DOI:** 10.1016/j.bioactmat.2025.03.024

**Published:** 2025-04-03

**Authors:** Jaibyung Choi, Suhyeon Shim, Jiwoo Shin, Ahhyun Lee, Jaan Strang, Tobias Braun, Reto Naef, Hyungil Jung

**Affiliations:** a Department of Biotechnology, Yonsei University, 50 Yonsei-ro, Seodaemun-gu, Seoul, 03722, Republic of Korea; b Program in Integrative Biotechnology, Yonsei University, 85 Songdogwahak-ro, Yeonsu-gu, Incheon, 21983, Republic of Korea; c JUVIC Inc., 272 Digital-ro, Guro-gu, Seoul, 08389, Republic of Korea; d TOPADUR Pharma AG, Grabenstrasse 11A, Schlieren, 8952, Switzerland

**Keywords:** Hydrogel-forming microneedle, Suprachoroidal space, Candlelit, Ocular drug delivery, Posterior eye segment

## Abstract

The suprachoroidal space (SCS), which exists between the sclera and choroid, offers a promising delivery route to the posterior segment of the eye (PSE) and is integrated with hollow microneedles (HMNs) for minimally invasive delivery. However, HMNs are limited by backflow owing to their narrow channel. Therefore, this study proposes a biocompatible SCS-inducing hydrogel-forming microneedle (SI-HFMN) with a specially designed candlelit shape that swells to separate the sclera from the choroid. The induced SCS provides a route for delivering loaded drugs to the PSE upon application. The optimized formulation of 20 % (w/w) poly(methyl vinyl ether-alt-maleic acid) (PMVE/MA) crosslinked with 7.5 % (w/w) polyethylene glycol (PEG) possesses sufficient mechanical strength (5.1 ± 0.7 N) to penetrate both the sclera and swell by 356 ± 28 %, to mechanically stimulate SCS formation. The formulation also recorded a drug absorption amount of 101 ± 9 μg/mg of hydrogel. Furthermore, *in vitro* and *ex vivo* experiments demonstrated the ability of the SI-HFMN to deliver drugs to the PSE via the formed SCS. Thus, this system offers an innovative method for drug delivery to PSE by inducing SCS formation.

## Highlights

• SI-HFMN is a platform designed for targeted drug delivery to the posterior segment of the eye.
• The technology involves a microneedle that induces suprachoroidal space formation with minimal invasiveness.
• The microneedle is made of biocompatible materials that are safe for use in the human body.
• Upon insertion, the microneedle swells to both mechanically stimulate the SCS and release the drug simultaneously.

## Graphical abstract

**Figure:**
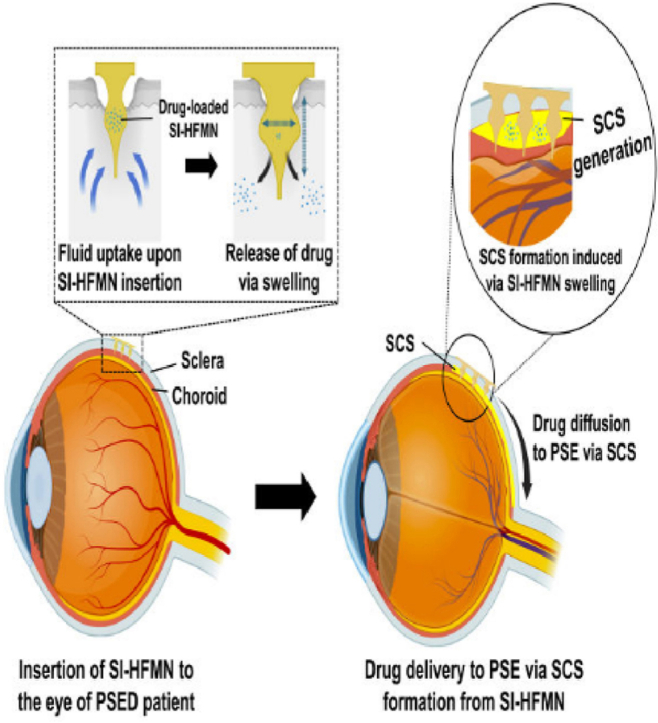

## 1 Introduction

Overall increased life expectancy and contemporary lifestyle factors led to the rise of modern chronic diseases, causing ocular-related complications in the posterior segment of the eye (PSE) [1,2]. These complications are characterized by abnormal growth or degeneration of blood vessels in the retina, leading to the degeneration of retinal pigment epithelium cells, which may cause impaired or complete loss of vision and pain from increased ocular pressure [3,4]. Intravitreal injection of drugs, where the administered drug diffuses through the vitreous humor and eventually reaches the PSE, is used to treat these symptoms [5]. However, this process is invasive and may cause severe complications, such as endophthalmitis, leading to pain and temporary vision impairment [6]. Moreover, the injected dose undergoes rapid clearance and random diffusion, reducing pharmaceutical efficacy [7].

The suprachoroidal space (SCS), a potential space between the sclera and choroid normally sealed due to intraocular pressure and the eye's fiber network, is a promising route to overcome these limitations and efficiently target PSE [8]. Fluid solution injection or mechanical penetration of the sclera using a catheter or hypodermic needles can separate the sclera and choroid for SCS [[9], [10], [11]]. Upon injection, the drug diffuses circumferentially to the PSE, because the SCS is located throughout the globe of the eye, achieving pharmacokinetic efficiency [9]. Despite these advantages, conventional methods to access the SCS remain invasive and require precise application by a skilled technician owing to difficulties in accessing the SCS, all of which may be addressed via a new drug delivery system [9,12].

Microneedles (MNs) are micron-sized needles that have been researched as drug delivery systems (DDS) owing to their minimally invasive nature, ease of application, and delivery efficiency [13,14]. Among these, hollow MNs (HMNs) created from stainless steel, gold, or glass, where each MN has a hollow opening for drug administration, have been widely used in SCS applications [14]. The most successful case of integrating HMN with SCS is Clearside Biomedical Inc.’s SCS Microinjector®, an HMN-based ocular DDS currently undergoing clinical trials [15]. Furthermore, SCS injection via microneedles has recently been proven to be a safe and minimally invasive delivery route for triamcinolone acetonide in the Phase III PEACHTREE trial, earning approval from the U.S. Food and Drug Administration [16]. However, despite advancements in their development, HMN faces the inevitable limitation of backflow in the HMN owing to its narrow channel, inducing clogging and potentially inconsistent drug delivery [14,17,18]. Ultimately, there may be a need to develop a safer and more efficient DDS for SCS.

Hydrogel-forming MNs (HFMNs) are an attractive alternative for overcoming these issues. HFMNs are sturdy, biocompatible polymeric MNs that swell upon absorbing fluids after application, releasing drugs from the polymer matrix [19]. The swellability of the HFMNs offers a versatile use for application in drug delivery, allowing the MNs both to interlock in the applied tissue as well as deliver the drugs in a sustained manner [20]. Therefore, researchers have applied HFMNs to ocular drug delivery primarily to utilize their sustained release property, which prevents repetitive injection into the eye and lowers invasiveness [21]. Lee et al. reported an implantable HFMN system that rapidly released a hydrogel-formulated drug in the vitreous humor for sustained release, whereas Amer et al. developed self-adhesive HFMNs for sustained ocular drug delivery to target the vitreous humor [21,22]. Both technologies targeted PSE via sustained drug delivery in the vitreous humor. However, targeting the vitreous humor relies on random diffusion for delivery to the PSE, and thus the limitation of targeted drug delivery via an HFMN system to the PSE remains unresolved [7]. Furthermore, conventional cone- or pyramid-shaped HFMNs swell vertically in a relatively less controlled manner, making it challenging to specifically and efficiently target and localize delivery in the SCS, as the SCS requires mechanical stimulation at a precise depth to induce its formation [9]. Nevertheless, appropriately designed and developed HFMNs may have the potential to target SCS for drug delivery to PSE.

Therefore, we propose an innovative SCS-inducing HFMN (SI-HFMN) to enhance the efficiency of drug delivery to PSE. Each SI-HFMN was specifically designed in a candlelit shape, where its thickest segment (‘head’) was measured to reach the depth where SCS may be induced, and the head segment swelled to provide maximum mechanical stimulation to separate the sclera and choroid and induce SCS formation while releasing its loaded drugs. The SI-HFMN formulation was optimized to 20 % (w/w) poly(methyl vinyl ether-alt-maleic acid) (PMVE/MA) crosslinked with 7.5 % (w/w) polyethylene glycol (PEG). Mechanical characteristic analysis and swelling studies revealed that the optimized SI-HFMN possessed a mechanical strength of 5.1 ± 0.7 N with a swelling percentage of 356 ± 28 %, allowing the SI-HFMN to penetrate the sclera, swell inside, and induce SCS formation. The drug absorption studies verified that the SI-HFMN could absorb drugs in its matrix at a ratio of 101 ± 9 μg/mg of hydrogel. Finally, an *ex vivo* study on porcine cadaver eye confirmed the ability of the SI-HFMN to penetrate the sclera, swell to induce SCS formation, and deliver the loaded drug to the PSE via the SCS. Therefore, this system provides a groundbreaking method for drug delivery to PSE via forming SCS through the swelling mechanism of SI-HFMN and serves as a platform for ocular drug delivery.

## 2 Materials and methods

### 2.1 Fabrication of PDMS mold

Hyaluronic acid (HA, 32 kDa, Bloomage Freda Biopharm, Jinan, China) was homogenized with deionized water (DW) to make 20 % (w/v) and 60 % (w/v) solutions using a paste mixer (PDM-300, KMtech, Gyeonggi, South Korea) at 164×*g* for 45 min. To fabricate the base layer, 20 % (w/v) HA solution was dispensed on a plasma-treated metal plate using a dispenser (SHOTmini 100S, Musashi Engineering, Tokyo, Japan). The metal plates were flipped onto a 300 μm-tall frame, and the HA droplets were self-shaped into an hourglass via g-force. After 3 h, the metal plates were flipped back to form the base layer, and a 60 % (w/v) HA solution was dispensed onto the formed base layers. The droplets were formed into a candlelit microneedle shape using centrifugal lithography to create a final master mold [23]. The diameters and heights of the master mold were measured using a brightfield microscope (M165FC, Leica, Wetzlar, Germany). To fabricate the candlelit-shaped cavity containing polydimethylsiloxane (PDMS) mold, the pre-compound solution was prepared by mixing Sylgard 184A (prepolymer, Sigma-Aldrich, St. Louis, MO, USA) and Sylgard 184B (curing agent, Sigma-Aldrich, St. Louis, MO, USA) in a 10:1 wt ratio. The solution was poured over the previously fabricated master mold in a reservoir and centrifuged at 300×*g* for 15 min in a vacuum to remove air bubbles. Then, the mixture compound was cured at 60 °C for 4 h, and the master mold was removed to form a PDMS mold.

### 2.2 Fabrication of SI-HFMN

PMVE/MA (1980 kDa, Sigma-Aldrich, St. Louis, MO, USA) powder was mixed with PEG (10 kDa, Sigma-Aldrich, St. Louis, MO, USA) granules and DW using a paste mixer (PDM-300, KMtech, Gyeonggi, South Korea) at 164×*g* for 1 h. The solutions were subsequently centrifuged at 2360×*g* for 15 min to remove the air bubbles. Centrifugal casting was used to fill the micro-cavities of the mold with the solution. Initially, 500 μL of the solution was cast onto the mold surface and centrifuged at 3082×*g* for 15 min. An additional 500 μL of the same solution was added as a backing film layer and centrifuged at 48×*g* for 5 min. Following centrifugation, the solution-containing molds were dried for 48 h at constant humidity (24 %) using a dehumidifier (ADH-EV60, HORUSBENNU, Seoul, Korea). Afterward, the molds were cross-linked at 80 °C for 24 h in an oven (Lab Companion, JEIO Tech, Seoul, Korea) to create the SI-HFMN. The diameters and heights of the SI-HFMN were measured using a brightfield microscope.

### 2.3 Biocompatibility assessment of SI-HFMN

The biocompatibility of the SI-HFMN was assessed via live/dead assay and 3-(4,5-dimethylthiazol-2-yl)-2,5-diphenyltetrazolium bromide (MTT) assay. The assays were conducted on human retinal pigment epithelial cells (ARPE-19, ATCC, Manassas, VA). The live/dead assay was conducted according to the manufacturer's instructions for the Viability/Cytotoxicity Assay Kit for Animal Live & Dead Cells (Biotium, Fremont, CA). The cells were initially cultured in Dulbecco's Modified Eagle Medium (DMEM/F-12) and 10 % fetal bovine serum (FBS, Sigma-Aldrich, St. Louis, MO, USA) added with 1 % (w/v) penicillin/streptomycin (Thermo Fisher Scientific, Walthman, MA, USA) at 37 °C and a CO_2_ concentration of 5 % until 80 % cell growth was achieved. Then, the cells were seeded at a density of 2 × 10^4^ cells per well in a 96-well microplate (SPL Life Sciences, Gyeonggi, South Korea) and incubated for 2 days. Next, the SI-HFMNs were disinfected by UV light and soaked in 5 mL of DMEM cell culture medium. The resulting samples were filtered through 0.2 μm syringe filters (Hyundai Micro, Gyeonggi, South Korea). The culture medium was then removed from the 96-well plates and replaced with 200 μL of the filtered samples, followed by incubation for 24 h. Subsequently, the cells were rinsed twice with PBS and incubated with a solution containing 2.5 μM calcein-AM and 2.5 μM ethidium homodimer III for 40 min at room temperature. Finally, the fluorescence staining of the cells was observed using a fluorescence microscope (M165FC, Leica, Wetzlar, Germany). For positive and negative controls, an untreated cell culture medium group and dimethyl sulfoxide (DMSO, Sigma-Aldrich, St. Louis, MO, USA)-treated groups were chosen, respectively.

For the MTT assay (VWR, West Chester, PA), the cells were cultured using the same method until 80 % cell growth and seeded at a density of 2 × 10^4^ cells per well in a 96-well microplate (SPL Life Sciences, Gyeonggi, South Korea) and incubated for 2 days. The SI-HFMNs were also disinfected and soaked in 5 mL of DMEM for 24 h, then filtered through 0.2 μm syringe filters (Hyundai Micro, Gyeonggi, South Korea). Afterward, 200 μL of the media were replaced with 200 μL of the filtered samples and incubated for 24 h. Then, 10 μL of MTT reagent was added to each well at a final concentration of 0.5 mg/mL and incubated for 4 h. Following removal of the media, 100 μL of DMSO and formazan were added and incubated for 2 h in the dark, and then absorbance at 570 nm was recorded to calculate cell viability. For positive and negative controls, an untreated cell culture medium group and DMSO-treated groups were chosen, respectively.

### 2.4 ATR-FTIR analysis of SI-HFMN

A Fourier transform infrared (FTIR) spectrometer (VERTEX 70, Bruker, MA, USA) with an attenuated total reflection (ATR) attachment was used to derive the absorbance of each SI-HFMN and the powder in the wavenumber range of 4000–650 cm^−1^ with a resolution of 4 cm^−1^. The samples were scanned 32 times against the background, and the values were normalized and plotted.

### 2.5 Physical properties of SI-HFMN

The fracture forces of each SI-HFMN array were evaluated using a force analyzer (Z0.5 TN, Zwick/Roell, Ulm, Germany). A single MN was separated from the array, and the pressure was applied using a probe programmed to displace vertically towards the MN at a speed of 3.6 mm/min. The force at which the probe deformed the MN tip was recorded. The penetration ability of the SI-HFMN arrays in the ocular environment was analyzed using an *in vitro* sclera-mimicking model. The model was prepared by mixing Sylgard 184A and Sylgard 184B in a 20:1 ratio to prepare a PDMS pre-compound solution. The pre-compound solution was degassed by centrifugation at 1734×*g* for 5 min and poured into a homemade mold, and was subsequently cured at 60 °C for 4 h to form the sclera-mimicking PDMS model. SI-HFMN arrays were then applied to the model and imaged using a brightfield microscope.

### 2.6 Swelling analysis

The SI-HFMN arrays were initially weighed (*m*_*0*_) using a scale (Discovery DV125CD, Ohaus, NJ, USA) and placed in individual Petri dishes containing 5 mL of 1X PBS (Sigma-Aldrich, St. Louis, MO, USA). The arrays were removed from the Petri dishes at the predetermined time points, gently dried using a paper towel, and weighed (*m*_*t*_). The following equation was used to derive the swelling percentage (*S%*) of each array at each time point: *S*(%) = (*m*_*t*_-*m*_*0*_)/*m*_*0*_ × 100 %. The variable *m*_*0*_ is the initial SI-HFMN array weight. A *t* vs. *S%* graph was plotted.

The swelling curve data were further applied to pseudo-second-order kinetics analysis, and the *t* vs. *t/S* graphs were plotted using the following equation [24]:

$$\frac{t}{S}=\frac{1}{k_{s}{S_{\infty }}^{2}}+\frac{1}{S_{\infty }}t$$

*S* is the *S%* at each time point, and *k*_*s*_ is the swelling rate constant. The slope and *y*-intercept were derived from the line of best fit of the *t* vs. *t/S* plot. Furthermore, the hydrogel was redried in an oven at 80 °C overnight to revert to its xerogel form. The mass of the xerogel was weighed (*m*_*x*_) and used to calculate the equilibrium water content (*EWC*) and gel fraction (*GF*) of the gels using the following equations: *EWC*(%) = (*m*_*∞*_-*m*_*x*_)/*m*_*x*_ × 100 % and *GF*(%) = *m*_*x*_*/m*_*0*_ × 100 %.

The mass at equilibrium swelling (*m*_*∞*_) was calculated empirically. The EWC and GF in DW were also measured and calculated using the same method. The porosity of each SI-HFMN array was analyzed using the solvent displacement method, with ethanol as the solvent [25]. Each array was then placed in absolute ethanol (Duksan, Gyeonggi, Korea) for 4 h. The mass of the ethanol-treated array (*m*_*EtOH*_) and initial mass (*m*_*0*_) were weighed and used to calculate the porosity (*φ*) using the following equation: *φ* = (m_EtOH_ – m_0_)/(V_T ×_ ρ_EtOH_).

*V*_*T*_ is the volume of the total hydrogel, and *ρ*_*EtOH*_ is the density of ethanol, which is 789 kg/m^3^, or 0.789 g/cm^3^.

The volume of each SI-HFMN was calculated at each time point (*V*_*t*_) by capturing high-resolution images of the MN using a brightfield microscope (M165FC, Leica, Wetzlar, Germany), and its height and diameter were measured at several intervals. Setting the tip section as the origin (0,0), the local maximum for each interval was plotted in the 1st quadrant of the X-Y plane (*x, f(x)*), and the curve of the best fit was determined as *f(x)* for each MN. The *V*_*t*_ was calculated using the following equation:

$$V_{t}=\pi \int_{x_{n}}^{x_{n+1}}{\left(f\left(x\right)\right)}^{2}d\left(x\right)$$

The integration intervals [*x*_*n*_*, x*_*n+1*_] represent each height interval, and the total sum reflects the theoretical volume of the MN at each time point.

### 2.7 Scanning electron microscopy

Scanning electron microscopy (SEM) analysis was performed using a field emission scanning electron microscope (FE-SEM, JSM-7610F-Plus, JEOL, Tokyo, Japan) to evaluate the surface characteristics of the SI-HFMN arrays before and after 3 h of swelling in 1X PBS. The swollen SI-HFMN samples were dehydrated in an oven for 3 h before imaging. The secondary electron imaging (SEI) mode was used, with a working distance of 38 mm and 10.0 kV of voltage.

### 2.8 Drug absorption analysis

First, a 1 % (w/v) lidocaine-HCl (Li-HCl, Sigma-Aldrich, St. Louis, MO, USA) solution was prepared. The SI-HFMN arrays were then added to the prepared Li-HCl solution and left to swell for 24 h. Subsequently, the arrays were removed from the Li-HCl solution and air-dried at room temperature for 24 h. The deswollen arrays were imaged using a brightfield microscope, added to each 4 mL of 1X PBS reservoir, and left to swell again for 24 h. Finally, 200 μL aliquots were extracted from each reservoir and analyzed using reverse-phase high-performance liquid chromatography (HPLC, Waters, Milford, MA, USA) with a C18 column (150 mm × 4.6 i. d., Cosmosil 5C18-AR-II, Nacalei Tesque Inc., Kyoto, Japan). The mobile phase consisted of 0.1 % (v/v) trifluoroacetic acid (TFA, Sigma-Aldrich, St. Louis, MO, USA) in distilled water (DW) and 0.1 % (v/v) TFA in acetonitrile (ACN, Sigma-Aldrich, St. Louis, MO, USA), flowing at a 30:70 ratio at 1.0 mL/min under isocratic conditions. The injection volume was 10 μL, and Li-HCl was quantified at a wavelength of 254 nm. The following equation was used to calculate the amount of absorption: Absorbed amount (μg/mg) = *C*_*Li-HCl*_/*M*_*x.*_ *C*_*Li-HCl*_ is the concentration of the absorbed Li-HCl solution, and *M*_*x*_ is the mass of each SI-HFMN array.

The permeability coefficient (*P*) was derived from the following equation using empirical values [26]: ln[1-(2*C*_*t*_*/C*_*0*_)] = (*-*2A/V) × (*Pt*). *C*_*t*_ is the drug concentration absorbed inside the SI-HFMN array 24 h after submersion and removal, and *C*_*0*_ is the initial bulk solution concentration (10 mg/mL). *V* is the drug solution volume, and *A* is the area of the SI-HFMN array, or the area of drug permeation. The solute partition coefficient (*K*_*d*_) was calculated from the experimentally obtained values using the following equation: *K*_*d*_ *= C*_*m*_*/C*_*24*_.

*C*_*m*_ is the absorbed drug concentration in the SI-HFMN array, and *C*_*24*_ is the remaining drug concentration in the reservoir 24 h after SI-HFMN array submersion. The calculated *P* and *K*_*d*_ values were used to calculate the diffusion coefficient (*D*) using the following equation: *D = PL/K*_*d*_. *L* is the SI-HFMN array thickness after 24 h in the drug solution.

The above parameters were also calculated for Nile red absorption within the SI-HFMN arrays, where a 0.1 % (w/v) Nile red (Sigma-Aldrich, St. Louis, MO, USA) solution was also absorbed using the same method. The concentrations were measured using a plate reader (PerkinElmer, Waltham, MA, USA) at an absorbance of 560 nm, and the *P, D, and K*_*d*_ values were calculated using the equations above.

### 2.9 Drug release analysis

The drug release kinetics of the fabricated SI-HFMN arrays were analyzed via a simple release analysis system. The drug release kinetics of the fabricated SI-HFMN arrays were analyzed via a simple release analysis system. The SI-HFMN was initially absorbed with Li-HCl using the swell/deswell method. Afterward, it was placed in a chamber filled with 14 mL of 1X PBS and set to 37 °C. The chamber was rotated at a constant speed of 200 rpm, allowing the arrays to be constantly floating. Then, 1 mL aliquots were extracted from the chamber at predetermined time points and replaced with an equal volume of PBS. The extracted aliquots were analyzed by HPLC, as described in the previous section, and the losses from the exchange of Li-HCl with PBS were derived from the permeability data. The percentage concentration of the drug released at each time point (*C%*) was calculated by dividing the concentration of the drug released at each time point (*C*_*t*_) by the relative maximum release concentration (*C*_max_), where *C*_max_ is the total drug concentration at 24 h. The cumulative percentage of drug release was plotted against time.

The drug release kinetics of the SI-HFMN were modeled using Tao Lu's kinetic model for the ultra-fast release of drug delivery systems, as follows [27]:

$$\frac{Q_{t}}{Q_{24\;h}}=y=\frac{k\times t}{1+a\times t}$$

*Q*_*t*_ is the concentration of the drug released at each time point, *Q*_*24h*_ is the cumulative drug concentration released at 24 h, *k* is the release rate constant, and *a* is an empirical parameter. The equation can be rewritten as

$$\frac{1}{y}=\frac{Q_{24\;h}}{Q_{t}}=\frac{1}{k}\times \frac{1}{t}+\frac{a}{k}$$

Finally, a 1/*t* vs. *Q*_*24*_*/Q*_*t*_ curve was plotted, and linear regression analysis was performed to derive the *k* value from the slope of the line of best fit.

### 2.10 In vitro swelling effect on tissue model

The agarose powder (Invitrogen, Carlsbad, CA, USA) was added to DW and heated to 100 °C using a microwave for 2 min. The homogenous solution was then poured into a homemade mold and cured in a refrigerator for 10 min, after which it was separated from the mold to obtain the *in vitro* tissue model. The SI-HFMN arrays, loaded with 0.01 % (w/v) Rho B using the swell/deswell method, were applied to the tissue model and imaged at 30 and 60 min. A fluorescent stereomicroscope was also used to generate fluorescent images of the SI-HFMN-treated gels. Finally, the ImageJ software (National Institutes of Health, Bethesda, MD, USA) was used to obtain the relative fluorescence intensity.

### 2.11 Evaluation of *ex vivo* SCS formation by SI-HFMN

Before analysis, the *ex vivo* porcine cadaver eyes (CRONEX, Hwaseong, South Korea) were thawed for 30 min in PBS at 37 °C, and thoroughly wiped using paper towels. The SI-HFMN arrays were pressed onto the sclera of the dried porcine cadaver eye, and OCT images were recorded using an OCT probe (LabScope 2.0, Lumedica, Inc., Durham, NC, USA). Finally, drug delivery analysis in *ex vivo* SCS was performed by encapsulating the SI-HFMN arrays with a 0.1 % (w/v) Nile red solution using the swell/deswell method. The Nile red-absorbed SI-HFMN arrays were then applied to the *ex vivo* porcine eyes for 1 h and removed. After 1 h, the application area of the eye was imaged using a fluorescence microscope and sectioned using surgical scissors to obtain the cross-sectional areas. Cross-sectional images were obtained by fluorescence microscopy. A blank SI-HFMN array was also imaged before and after application to the *ex vivo* porcine cadaver eyes via a brightfield microscope.

### 2.12 Statistical analysis

The Student's t-test was used to compare differences between two groups. One-way analysis of variance (ANOVA) with Tukey's or Šidák's multiple comparison test was used to compare three or more groups. Statistical significance was set at P < 0.05. All statistical analyses were performed using GraphPad Prism 9 (GraphPad Software, San Diego, CA, USA). All data are presented as the mean ± standard error of the mean (SEM).

## 3 Results and discussion

### 3.1 Mechanism and design of the SI-HFMN

**Fig. 1:**
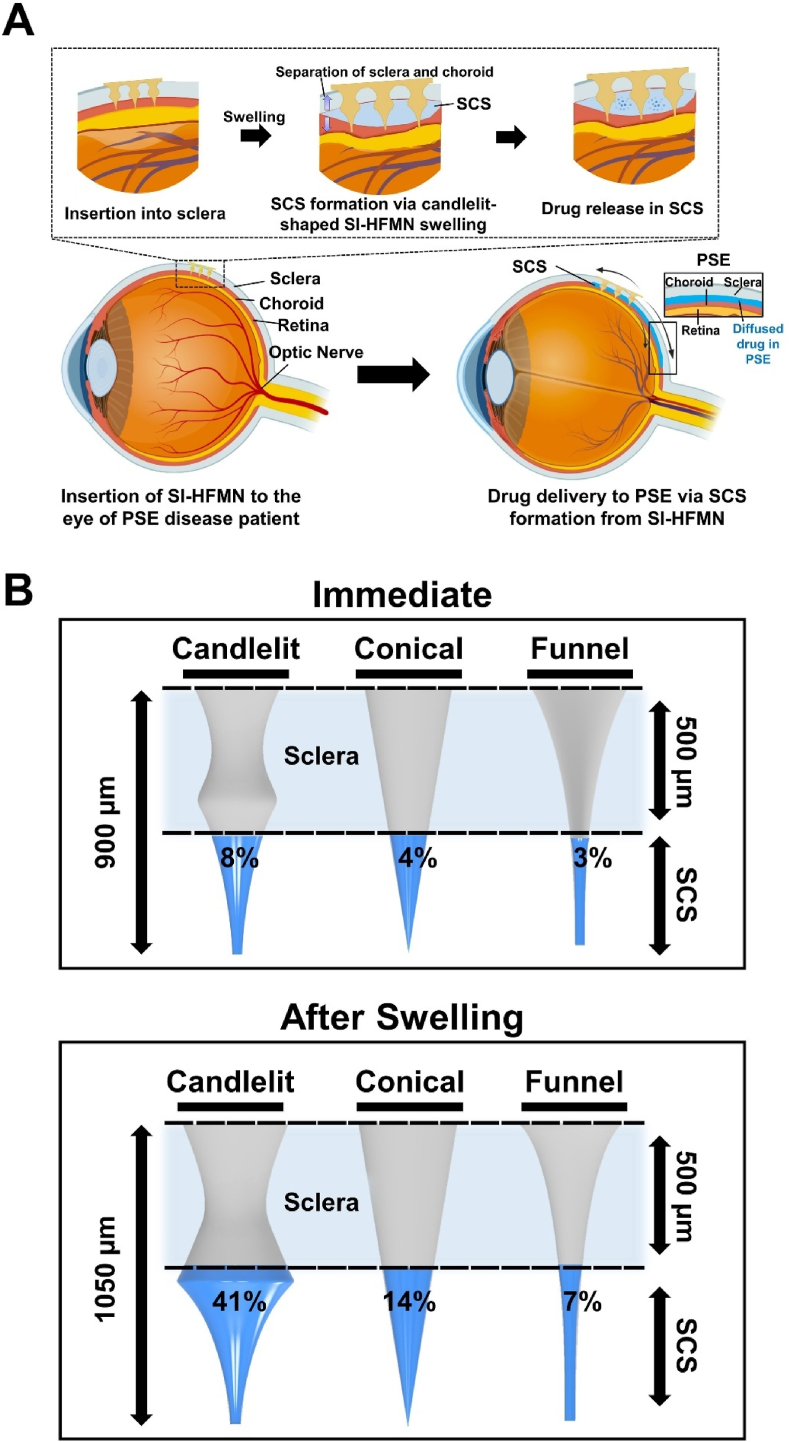
Design of suprachoroidal space-inducing hydrogel-forming microneedle (SI-HFMN) for delivery to the posterior segment of the eye (PSE). (A) Schematic illustration of the SI-HFMN mechanism. Upon insertion into the eye of a PSE disease patient, the SI-HFMN absorbs the surrounding fluid and swells, inducing SCS formation to generate an access route to the PSE and simultaneously release its absorbed drug. The released drug can then diffuse through the SCS to target the PSE. (B) Theoretical volume distribution of various MN shapes (top) immediately after insertion and (bottom) after swelling. The (left) candlelit-shaped MN, (middle) conical-shaped MN, and (right) funnel-shaped MN were designed to be 900 μm high, and 1050 μm high after swelling. Light blue areas between the dotted lines represent sclera width.

SI-HFMN utilizes the unique mechanism of a swellable HFMN array to induce SCS formation, thereby integrating the advantages of HFMN and SCS as drug delivery routes to PSE. A schematic overview of the HFMN mechanism is shown in Fig. 1A. Upon insertion of the drug-absorbed SI-HFMN in the eye of the PSE disease patient, the HFMNs absorbed the surrounding fluids and swelled, leading to an expansion in their polymer matrix, which acted as a mechanical stimulation for SCS formation and allowed the HFMN to release the loaded drug into the SCS [18].

Each MN was designed in a candlelit shape to maximize swelling volume at the depth where SCS may form (∼500 μm at the limbus), subsequently providing high mechanical stimulation at that particular depth for efficient SCS formation [28]. Fig. 1B illustrated this concept, where the MN volume was relatively higher in the ‘head’ of the candlelit-MN where the SCS was located, as opposed to other more conventional conical or funnel-type MN shapes where most of the volume was concentrated in their ‘base,’ far from the SCS location [29]. All MNs were set to 900 μm, long enough to penetrate the sclera at any portion of the eye and access the SCS [28]. We calculated the theoretical volume of each MN in the SCS after application, which is the most important concept, as a higher volume in the SCS would lead to more swelling, allowing increased SCS induction. In contrast to the volume of the MN inside the SCS immediately after insertion, the volume after swelling showed drastic differences between the candlelit-(41 %), conical-(14 %), and funnel-shaped (7 %) MNs, with the potential to increase even further with longer application times. We based our assumptions on multiple studies that reported increases in MN height and base diameters of ∼150 μm and ∼75 μm, respectively [[30], [31], [32]]. The candlelit-shaped SI-HFMN could thus maximize its mechanical stimulation via a high swelling volume to efficiently induce SCS formation. Additionally, candlelit-MN can interlock in the tissue, preventing detachment of the MN once inserted and eliminating potential drug delivery losses [33]. Therefore, the candlelit-shaped SI-HFMN provides an innovative method to efficiently induce SCS formation via mechanical stimulation through its maximized volume distribution and delivery of drugs to the PSE.

### 3.2 Fabrication and characterization of the SI-HFMN

**Fig. 2:**
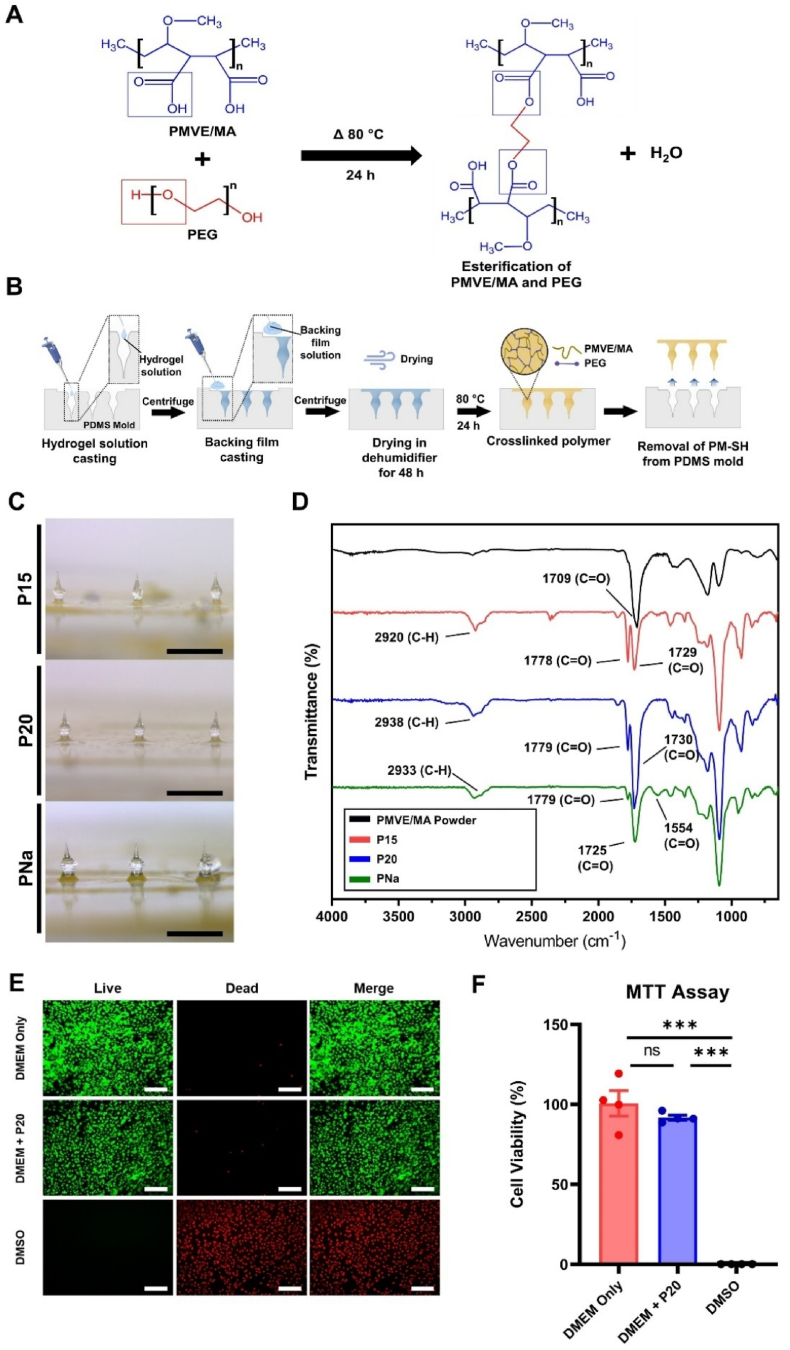
Fabrication and analysis of suprachoroidal space-inducing hydrogel-forming microneedle (SI-HFMN) arrays. (A) Proposed mechanism of esterification of poly(methyl vinyl ether-alt-maleic acid) (PMVE/MA) and polyethylene glycol (PEG). PMVE/MA and PEG were thermally processed, leading to the condensation reaction between the carboxylic acid group (blue box) of PMVE/MA and the hydroxyl group of PEG (red box) to form ester linkages. (B) Schematic of microneedle formation via micro-molding process. (C) Microscopic images of fabricated (top) P15, (middle) P20, and (bottom) PNa (scale bar: 1 mm). (D) Attenuated total reflection-Fourier transform infrared (ATR-FTIR) analysis of (black) PMVE/MA powder, (red) P15, (blue) P20, and (green) PNa. (E) Live/dead assay of P20 compared to positive control (DMEM Only, top) and negative control (DMSO) (scale bar: 200 μm). Green channel indicates live cells, red channel indicates dead cells. (F) Viability of P20-treated cells (middle, blue) compared to DMEM Only-treated (positive control, red) cells and DMSO-treated (right, black) cells. Data are represented as mean ± SEM (n = 4). ∗∗∗, P < 0.001, ns means not significant.

We designed SI-HFMN arrays with a 3 × 1 shape to minimize the application area and decrease the invasiveness to the eye during application. We selected PMVE/MA as the backbone polymer owing to its mechanical strength, biocompatibility, and ability to cross-link its functional groups with polyhydric alcohols such as PEG to plasticize the polymer via ester linkages and form a swellable hydrogel (Fig. 2A**)** [19,34]. We tested various compositions of previously published formulations, where the PMVE/MA:PEG ratio was set to either 2:1 or 8:3, with PMVE/MA 15 %-based SI-HFMN (P15) and PMVE/MA 20 %-based SI-HFMN (P20) crosslinked with 7.5 % PEG. Additionally, the modifying agent added group (PNa), which comprised 20 % PMVE/MA, was blended with 3 % sodium carbonate (Na_2_CO_3_), known for its induction of “super swelling” hydrogels [31,35]. The respective formulations are summarized in Table S1.

Fig. 2B illustrates the overall fabrication process of the candlelit-MN-shaped SI-HFMN arrays. Centrifugal casting was used to fill the hydrogel solution into a PDMS mold containing candlelit-shaped cavities, the preparation process of which is shown in Fig. S1. The solution-containing molds were dried and thermally processed to form the crosslinked SI-HFMN arrays, which were plasticized and therefore easily removed from the flexible PDMS mold. Fig. 2C shows the microscopic images of the formed P15, P20, and PNa, which duplicated the prepared master mold in Fig. S1 (n = 9, mean ± SEM) with a height of 748 ± 9 μm and head diameter of 354 ± 7 μm. Although previous studies revealed that 100 μm of the candlelit-MN remained uninserted suggesting an insertion depth of 650 μm, given that the SI-HFMN may swell, it may match the length consistent with the conventional MNs (∼700 μm) designed to target the SCS [29,36].

Subsequently, we analyzed the SI-HFMN arrays using ATR-FTIR spectroscopy to confirm the chemical shift after crosslinking using pure PMVE/MA powder as a reference. The PMVE/MA powder showed a strong peak at 1709 cm^−1^ corresponding to the carboxylic acid moiety's carbonyl group (CO) (Fig. 2D). In contrast, the SI-HFMNs displayed peaks at ∼1730 cm^−1^ and ∼1780 cm^−1^. The former indicates a chemical shift in CO resulting from crosslinking, whereas the latter represents carbonyl ester (CO_ester_) formation, symbolizing esterification. In addition, the peaks at ∼2930 cm^−1^ for the SI-HFMNs were attributed to the C-H bonds of the PEG, confirming crosslinking. Furthermore, the PNa exhibited a peak at 1554 cm^−1^ corresponding to the CO of the carbonate ion (CO_3_^2−^), validating the presence of Na_2_CO_3_ [31]. For the quantitative analysis, we compared the ratios of absorbance units (AU) between CO_ester_ and COOH in each group (CO_ester_/COOH) (Table S2). P15 had a ratio of 0.79, while P20 and PNa had a ratio of 0.54 and 0.27, respectively. Larger numbers indicate more CO_ester_ group formation and a higher crosslinking density. This was expected because the polymer-to-crosslinker ratio for P15 was 2:1, as opposed to those for P20 and PNa (8:3 for both), indicating that more of the polymer was crosslinked, leaving less residual unreacted COOH. Meanwhile, P20 had a higher crosslinking density than PNa because the Na^+^ ions presumably formed a sodium salt with the free acid of PMVE/MA, competitively reducing the number of bonding sites for the PEG [31]. Overall, all groups demonstrated an increase in the number of CO_ester_ groups, confirming successful crosslinking.

However, despite being able to reduce the number of unreacted COOH groups via crosslinking density, the residual COOH may potentially lower the pH of the hydrogel matrix, which may in turn affect the drug release or ionize the drugs absorbed within. Therefore, despite not potentially significantly affecting drug-polymer interactions for small molecules, the limitation of this formulation may have to be considered when applied to different drugs, especially those containing amino acids that may ionize into positively charged groups, in turn potentially interacting with the negatively charged acid groups of the PMVE/MA [37].

Additionally, we evaluated the cytotoxicity of the SI-HFMN arrays via live/dead assay and MTT assay, with P20 the representative formulation assessed. To qualitatively assess cytotoxicity, we incubated the cells with P20 treated media for 24 h and observed a minimal number of dead cells via the live/dead assay (Fig. 2E), indicated in the lack of red-stained cells in the DMEM + P20 groups. Furthermore, when we quantitatively analyzed cell viability, the DMEM + P20 showed an average of 92 ± 2 % viability compared to that of the DMEM only group (100 ± 8 %), with the difference not statistically significant (P = 0.41). Therefore, we were able to verify the biocompatibility of the formulation used in this study.

### 3.3 Fracture force and *in vitro* insertion analysis of SI-HFMN

**Fig. 3:**
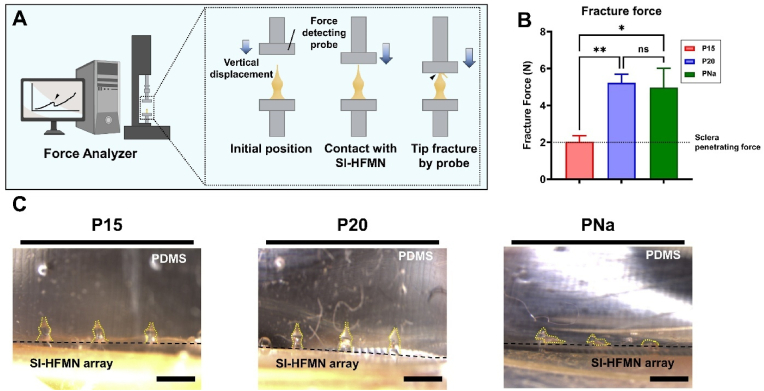
Evaluation of the physical property of each suprachoroidal space-inducing hydrogel-forming microneedle (SI-HFMN) formulation. (A) Schematic of fracture force analysis via force analyzer. Force-detecting probe travels vertically to reach and eventually fractures the tip of the SI-HFMN. Fracture force is recorded in the connected software. (B) Average fracture force per formulation. Black dotted line represents the minimum force needed to penetrate the sclera (2.07N). All data are represented as mean ± SEM (n = 10). ∗, P < 0.05; ∗∗, P < 0.01, ns means not significant. (C) Microscopic images of SI-HFMN application into PDMS-based sclera-mimicking model (scale bar: 1 mm).

As the sclera possesses remarkable mechanical strength owing to its dense collagen-rich fibrous structure designed to protect the eye and provide structural integrity, we investigated whether each SI-HFMN formulation could successfully penetrate the sclera by measuring the fracture forces using a force analyzer (Fig. 3A) [38]. An average of 2.07 N is required to penetrate the sclera and inject into the SCS using MN, represented by the dotted lines in Fig. 3B [9]. The respective average fracture forces (n = 10, mean ± SEM) were 2.1 ± 0.3 N, 5.1 ± 0.7 N, and 4.5 ± 1.2 N for P15, P20, and PNa, which were sufficient for sclera penetration. Furthermore, the sturdiness of the plasticized SI-HFMNs allowed successful removal of the microneedles without damage, achieving reproducibility in its fabrication process as well as ensuring prevention of potential fracturing of the SI-HFMN tips during insertion to the sclera.

While the fracture force suggests the ability of the SI-HFMN to penetrate the sclera, actual application in tissue models is needed to verify their mechanical characteristics. To confirm this, we applied SI-HFMNs to an *in vitro* sclera-mimicking model composed of a 20:1 ratio of PDMS, which was utilized to generate artificial eye models [39]. Notably, whereas the P15 and P20 arrays successfully penetrated the model and retained their candlelit-shaped morphology, the PNa group failed to puncture the PDMS surface despite possessing a higher fracture force than P15 (Fig. 3C). We assumed that the low crosslinking density of PNa may have contributed to its low mechanical force when applied to an actual tissue model because the model also consisted of multiple additional forces to overcome, such as friction and indentation forces, as opposed to the fracture force, which only involved resisting the force from a vertical probe [40]. These observations suggest the unreliability of PNa for further ocular applications, and we decided to remove this group from additional studies.

### 3.4 Swelling kinetics analysis of SI-HFMN

**Fig. 4:**
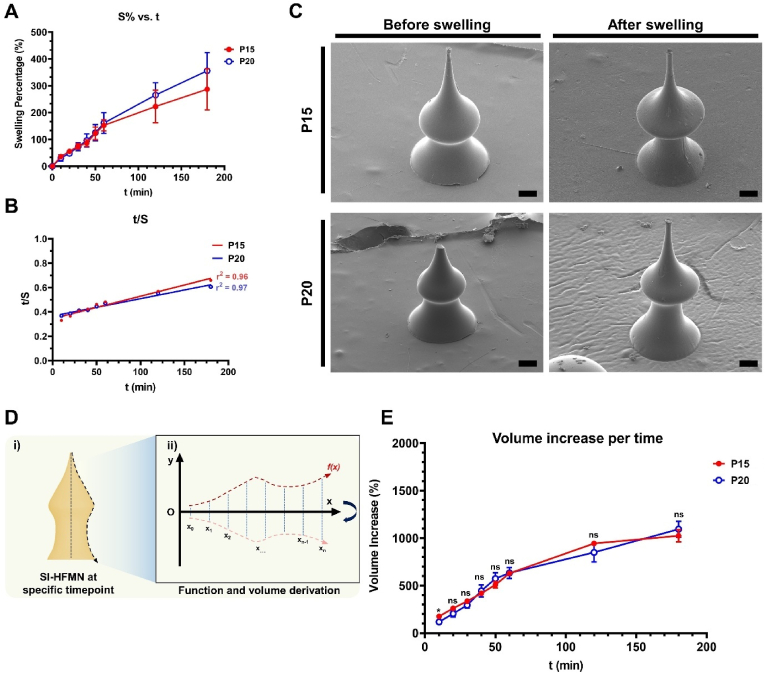
Suprachoroidal space-inducing hydrogel-forming microneedle (SI-HFMN) swelling kinetics analysis in phosphate buffered saline (PBS). (A) *S%* vs. t at each time point up to 180 min for (red) P15 and (blue) P20 (n = 6, mean ± SEM). (B) t/*S* vs. t curve for each formulation. Lines of best fit were derived via linear regression. (C) SEM images of each formulation before and after swelling (scale bar: 100 μm). (D) Overall process of SI-HFMN volume calculation per timepoint. (i) SI-HFMN were imaged at each time point and then (ii) converted into a Cartesian coordinate system whereby the volume was calculated using the integration of the derived function. (E) Volume increase (*V%*) per timepoint of each SI-HFMN formulation (n = 8, mean ± SEM). ∗, P < 0.05. ns means not significant.

Next, we analyzed the swelling kinetics of the remaining SI-HFMN formulation to predict its ability to form SCSs. Each array was soaked in phosphate-buffered saline (PBS) to mimic the human physiological environment, and the masses of the arrays were measured at each time point for up to 3 h, as multiple studies have reported the relative saturation of swelling within 200 min [41,42]. The swelling percentages (*S%*) of P15 and P20 were 287 ± 38 % and 356 ± 28 %, respectively (Fig. 4A, n = 6). Moreover, Fig. S2A shows the individual *S%* per time point, where P15 and P20 showed non-significant differences.

We further analyzed the swelling behavior by deriving the swelling parameters using pseudo-second-order kinetic equations, which are commonly used to model hydrogel interactions with water molecules leading to their swelling (Table 2) [43]. Fig. 4B shows the linearized form of *S%* in a *t* vs. *t/S* plot. The respective *r*^*2*^ values of 0.96 and 0.97 for P15 and P20 implied relative correlations toward a linear trend, suggesting that pseudo-second-order kinetics was a valid model for deducing the swelling parameters. Linear regression analysis revealed that P15 had a lower value of equilibrium swelling (*S*_*∞*_) (556 %) compared to that of P20 (714 %), confirming the *S%* trends. Moreover, P15 had a larger rate constant of swelling (*k*_*s*_) (9.41) than P20 (5.35) since P15 had a lower *S*_*∞*_, meaning it required less time to reach the equilibrium state. We also calculated the equilibrium water content (EWC) and gel fraction (GF) to verify the *S%* results. There was no significant difference (P = 0.94) in EWC_PBS_ between P15 (558 ± 21 %) and P20 (583 ± 11 %) as well as in GF_PBS_ (P = 0.27) between P15 (98 ± 1 %) and P20 (97 ± 1 %). We also observed a similar trend in EWC and GF in DW (Table S3) where P15 showed lower EWC_DW_ (170 ± 1 %) and higher GF_DW_ (98 ± 1 %) than those of P20 (193 ± 2 % and 94 ± 1 % for EWC_DW_ and GF_DW_, respectively). These results followed the pattern of high EWC and low GF corresponding to a higher *S%*. Additionally, the higher porosity (*Φ*) value of P20 (15 ± 2 %) compared to P15 (10 ± 2 %) indicated that lower crosslinking density led to a more porous hydrogel matrix and overall higher *S%*. In summary, given the superior strength and swellability of P20, it may be more suitable for SCS induction and drug release compared to P15. We also observed morphological changes in each formulation using qualitative SEM analysis (Fig. 4C). Both P15 and P20 appeared to show an elongated morphology from their original structures, implying similar swelling behaviors.

**Table 2:** Suprachoroidal space-inducing hydrogel-forming microneedle (SI-HFMN) swelling parameters in phosphate buffered saline (PBS) solution.

Formulation	S_∞_ [%]	k_s_^a^ [(mg/mg)/min]	EWC_PBS_ [%]	GF_PBS_ [%]	Φ [%]
P15	556	9.41	558 ± 21	98 ± 1	10 ± 2
P20	714	5.35	583 ± 11	97 ± 1	15 ± 2

a 10^−5^.

Furthermore, we analyzed the volume difference (*V%*) of SI-HFMN at each time point in PBS to visualize and predict the swelling morphologies of each formulation once applied to the eye, which is an aqueous environment. SI-HFMN arrays were imaged at predetermined time points (Fig. S2B), and the images were transformed into Cartesian coordinates, with the height of the SI-HFMN set as the *x*-axis and its surface set as the *y*-axis (Fig. 4D). The curve was integrated at each interval, and the resultant volume changes were displayed in Fig. 4E (n = 8, mean ± SEM). Consistent with the *S%* trend, the volume increase showed an overall higher increase for P20 (1094 ± 85 %) than P15 (1025 ± 64 %), although insignificantly (P = 0.60) different. Although a more detailed study is required to accurately deduce the contrasting correlation between *S%* and *V%*, the results suggest that while P20 showed slightly higher swellability, both P15 and P20 may not be very different in swelling behavior overall and thus require further optimization.

### 3.5 Drug absorption analysis of SI-HFMN

**Fig. 5:**
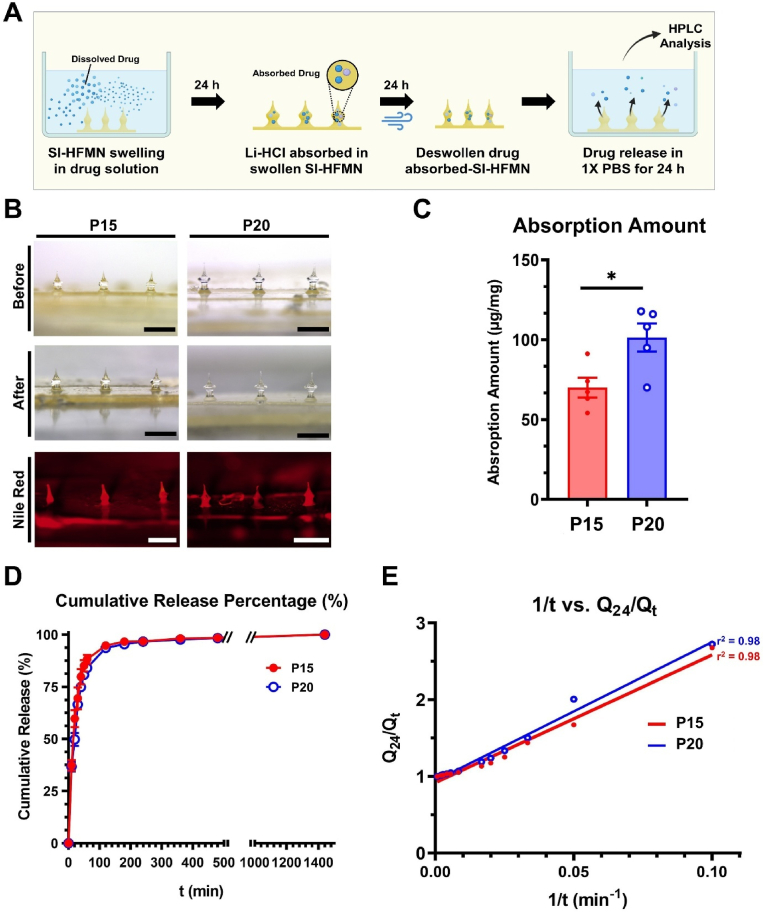
Drug absorption and release analysis of each suprachoroidal space-inducing hydrogel-forming microneedle (SI-HFMN) formulation. (A) Schematic illustration of the swell/deswell method and analysis. Each SI-HFMN array was treated in 1 % (w/v) Li-HCl solution for 24 h, then deswollen for 24 h via air drying. Deswollen SI-HFMN arrays were then submerged in 1X PBS solution for 24 h and subsequently analyzed via HPLC. (B) Microscopic images of each formulation (top) before and (middle) after swell/deswell method, and (bottom) Nile red absorption for drug absorption visualization (scale bar: 1 mm). (C) Li-HCl absorption amount (amount of drug/weight of array) for both formulations. (D) Graph of cumulative Li-HCl release percentage of up to 1440 min. (E) Release kinetic parameter analysis of SI-HFMN arrays. All data are represented as mean ± SEM (n = 5). ∗, P < 0.05.

To further deduce the optimal formulation for SI-HFMN, we analyzed the drug absorption amount for each SI-HFMN array to determine the average drug amount per weight of the array. The drug was directly loaded into the SI-HFMN matrix using the swell/deswell method, as the conventional method of integrating HFMN arrays with an external drug reservoir may not be suitable for ocular applications [35]. We used Li-HCl, a widely used analgesic drug applied to the eye, as a model drug [44]. Fig. 5A introduces an overview of the swell/deswell and drug absorption amount analysis. Each SI-HFMN array, with an average mass of 213 ± 8 mg and 260 ± 7 mg for P15 and P20, respectively, was swollen in 4 mL of a 1 % (w/v) Li-HCl solution, de-swollen, and re-swollen to release the absorbed Li-HCl for analysis. This method loads the drugs into the hydrogel matrix post SI-HFMN fabrication, avoiding potential drug degradation at high crosslinking temperatures if the drug was to be directly mixed with the hydrogel pre-compound solutions [45]. The formulations were immersed in the drug solution for 24 h, providing adequate time for equilibrium swelling to enable enhanced drug absorption within the matrix [46,47].

Fig. 5B shows that the morphologies of P15 and P20 did not change significantly after the absorption. Furthermore, both formulations could absorb the hydrophobic Nile red inside their matrix, confirming the viability of this method for loading hydrophobic drugs [48]. Additionally, the respective average fracture forces for P15 were 4.4 ± 0.4 N and 5.9 ± 0.8 N for P20 after drug absorption, which sufficiently overcome the minimum force of 2.07N required for sclera penetration (Fig. S3, n = 6, mean ± SEM). We observed an unexpected increase in the fracture force of SI-HFMN post-absorption. Li-HCL may have entered the swollen polymer matrix, displacing the water molecules, which may have led to a dryer and stiffer polymer matrix when the swelling is relieved. Nevertheless, the mechanical forces of both formulations were not affected by the swelling/deswelling method and were suitable for scleral penetration.

We then derived the drug absorption amount per unit weight of the array for each formulation, as indicated in Fig. 5C. P20 showed a higher absorption amount (101 ± 9 μg/mg) than that of P15 (70 ± 6 μg/mg) due to sparser crosslinking density, leading to a larger area for the drugs to diffuse into. To confirm this observation, we calculated various drug absorption parameters (Table 3). The higher solute permeability coefficient (*P∗*; ∗ = 10^−6^), solute partition coefficient (*K*_*d*_), and diffusion coefficient (*D∗*; ∗ = 10^−6^) value of the P20 group suggested that more of the Li-HCl could diffuse randomly inside the swollen polymer matrix, leading to the higher absorption amount. The drug absorption parameters for Nile red in the different formulations of SI-HFMN were also calculated (Table S4), and overall showed a similar trend to Li-HCl where P20 showed higher *P* and *D* as well as similar *K*_*d*_ than P15, implying that absorption kinetics behave similarly in hydrophobic drugs as well.

**Table 3:** Absorption and release kinetic parameters for Li-HCl in the suprachoroidal space-inducing hydrogel-forming microneedle (SI-HFMN) arrays.

Formulation	P^a^ [cm/s]	K_d_	D^a^ [cm^2^/s]	k^b^ [min^−1^]
P15	10 ± 1	0.10 ± 0.01	7.4	6.0 ± 0.7
P20	18 ± 1	0.18 ± 0.02	9.7	5.6 ± 0.6

a 10^−6^.

b 10^−2^.

The swelling/deswelling method correlated with the *S%* results, as the higher swellability of P20 led to an enhanced drug absorption efficiency. However, as the empirically derived absorption parameters were specific to Li-HCl, each parameter has to be individually derived if other drugs or solutes were to be used in the future.

### 3.6 Drug release analysis of SI-HFMN

We evaluated the drug release of different SI-HFMN formulations to further derive the optimal formulation for SCS induction and drug delivery to the PSE. Li-HCl absorbed SI-HFMN arrays were submerged in a PBS solution, and aliquots were extracted every 10 min for 60 min, and then up to 1440 min (24 h). The cumulative release percentage was graphed in Fig. 5D (n = 5, mean ± SEM), where the cumulative amount at 24 h was set to 100 % and each time point was the relative percentage released. Both groups showed an initial burst release up to 120 min with P15 and P20 releasing 94.8 ± 0.7 % and 93.7 ± 0.6 % of their respective absorbed amount with a release rate at 120 min of 0.78 %/min for both groups, calculated from the slope of the graph. Considering that the eye may not be suitable for the long application time of SI-HFMN, a fast drug release rate may be ideal for less invasive delivery to the PSE, as the required wearing time may be drastically shorter.

Additionally, we obtained the Li-HCl release parameters of the SI-HFMN arrays using Tao Lu's kinetic model for an in-depth analysis of the ultra-fast release trends [27]. The linearized graphs (Fig. 5E) for both P15 and P20 showed an *r*^*2*^ value of 0.98, suggesting the viability of this model. The slopes of the graphs were used to calculate the release rate constant (*k*∗∗; ∗∗ = 10^−2^), and the resulting values were 6.0 ± 0.7 and 5.6 ± 0.6 min^−1^ for P15 and P20, respectively (Table 3). Although P15 had a higher *k*∗∗ value, the insignificant (P = 0.56) difference between the two groups implied that P20 may be more appropriate for ocular applications because of its superior mechanical strength and similar drug delivery efficiency. However, the release parameter was specific to Li-HCl and may have been affected by the chemical composition, molecular weight, and various factors of the drug. This is especially evident as, despite the initial slow swelling rate, both formulations were able to release over 93 % of the cumulative amount released. This may be due to the small molecular weight of the Li-HCl (270.801 g/ml) compared to that of the PMVE/MA (1980 kDa), which may have allowed a much faster release from the hydrogel matrix [49]. If a different larger drug, such as proteins or larger molecules, were to be investigated, the slow initial rate may pose as a limitation, and additional studies may need to be conducted to optimize the formulation according to the drug used. Thus, it is essential to derive individual drug-release parameters for other drugs for future purposes.

### 3.7 Evaluation of drug delivery to the PSE via SCS formation

To predict whether the swellability of SI-HFMN can induce SCS formation, we first evaluated the strength of each formulation's swellability using a 5 % (w/v) agarose gel, which is a widely used *in vitro* tissue model for drug delivery studies [50]. Rhodamine B (Rho B)-absorbed arrays were applied to the model, and the fracturing of the gels and the concurrent absorbed drugs released were recorded (Fig. S4A). Whereas P20 fractured and pushed the agarose gel (black dotted line) and released its absorbed drug, P15 failed to significantly affect the agarose gel, leading to minimal swelling and reduced drug release (Fig. S4B). The relative fluorescence intensity unit (RFU) was quantitatively deduced from the fluorescent image (Fig. S4C, n = 4, mean ± SEM). P20 showed at least 1.6 times higher RFU at each time point (Table S5), suggesting that the swellability of P20 had greater mechanical strength and allowed more efficient drug release than P15.

**Fig. 6:**
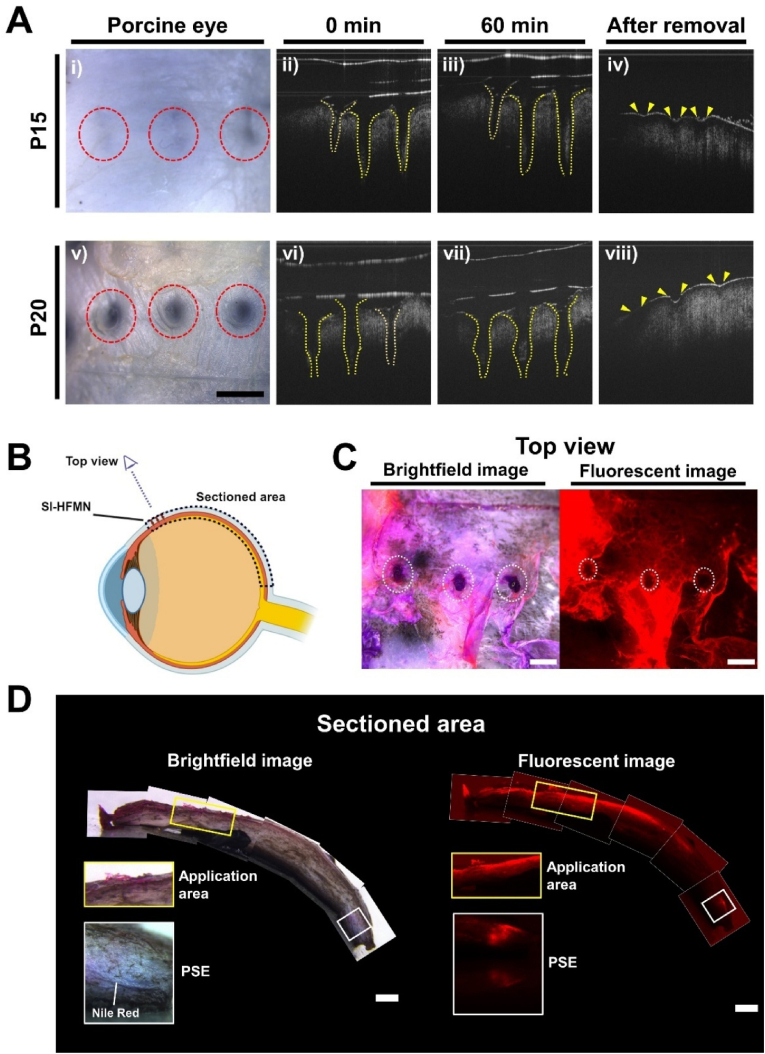
Evaluation of suprachoroidal space (SCS) formation and drug delivery of suprachoroidal space-inducing hydrogel-forming microneedle (SI-HFMN) on *ex vivo* porcine eye. (A) Real-time imaging of SI-HFMN application on *ex vivo* porcine eye. (i), (v) Brightfield image of porcine eye application for P15 and P20, respectively (scale bar: 1 mm). Optical coherence tomography (OCT) imaging of SI-HFMN application at (ii), (vi) 0 min, (iii), (vii) 60 min, and (iv), (vii) after removal of the arrays. (B) Schematic overview of the analytical process for SCS drug delivery in *ex vivo* porcine eye. After applying the Nile red-loaded MN arrays, the application area was imaged in the top and cross-section view. (C) Brightfield and fluorescent images of the top view of the Nile red-loaded MN applied area (scale bar: 1 mm). White dotted circles indicate successful MN insertion into the *ex vivo* porcine eye. (D) Brightfield (top) and fluorescent (bottom) view of SCS-induced drug delivery to the PSE. Yellow box represents the magnified view of the SI-HFMN application area. White box signifies a magnified view of the posterior segment of the eye (PSE).

We observed the induction of SCS formation via SI-HFMN in *ex vivo* porcine eyes using optical coherence tomography (OCT), a non-invasive ophthalmic imaging instrument commonly used to obtain 3D *in vivo* information [51]. Fig. 6A shows the brightfield and OCT images of the SI-HFMN applied to the *ex vivo* porcine eye, where P20 achieved full application (red circle) into the *ex vivo* porcine eye, as well as the ability to induce space formation inside the tissue via swelling (yellow dotted lines). Meanwhile, P15 showed a straight line of swelling, suggesting that only the tip of the SI-HFMN was inserted. Additionally, we observed minimal perforations on the eye post removal via the OCT (Fig. 6Aiv, vii), suggesting that the application process of the SI-HFMN is relatively minimally invasive. Considering recent studies have reported the safety of long-term presence of hydrogels inside the ocular space, reaching up to 1 month, the application time of 60 min for the SI-HFMNs may further imply the safety of this system [52]. Although further studies such as investigations of Young's Modulus are needed to additionally characterize the ability of SI-HFMNs to both insert and swell within the sclera, previous studies administering hydrogel formulations within the SCS of various animal models showed safe implantation for months, suggesting that the swelling of the SI-HFMN may have the potential to be minimally invasive and comfortably applied when translated to clinical settings [52,53].

Contrary to our *in vitro* results, where the respective *S%* and *V%* were approximately 170 % and 700 % for P20 at 60 min, its swelling in the actual *ex vivo* tissue was relatively minimal when referring to the OCT images. This was expected because our *in vitro* experiments were performed in a PBS solution, which possesses minimal resistance forces for the swelling of P20. In contrast, the dense fiber network of the sclera, which possesses high resisting forces, may have led to the lower swellability of the SI-HFMN [38]. Nevertheless, P20 was able to insert into the sclera and swell despite its resistance, subsequently altering the tissue structure and generating space. Therefore, we concluded that P20 has the potential to induce SCS formation via its swellability.

To confirm our observations, we evaluated the ability of SI-HFMN to deliver absorbed drugs to PSE via SCS induction, using Nile red as the drug surrogate. As P15 failed to penetrate the sclera, we chose P20 as the final formulation for evaluation. Fig. 6B shows an overview of the analysis process in which the Nile red-loaded SI-HFMN was initially applied 4 mm away from the limbus, which is the area for SCS formation induction [54]. The MN arrays were removed 1 h later, with the top view imaged immediately and subsequently sectioned for a cross-sectional view. Fig. 6C displays top-view images showing successful SI-HFMN insertion into the eye. To assess SI-HFMN breakage and potential safety issues, we also applied a blank SI-HFMN array on *ex vivo* porcine cadaver eye (Fig. S5), where the images post application showed an intact array without tip or hydrogel fracturing, implying its safety in application. Furthermore, the fluorescent image of the sectioned area showed that the dye diffused through the space formed near the sclera into the PSE, as represented by the trail of the red fluorescent signal (Fig. 6D). Moreover, the magnified section of the PSE in the brightfield image shows visible Nile red dye, which was confirmed by its bluish-purple color. We also did not observe fluorescent signal diffusion into the choroid area of the eye, from which we assumed that SI-HFMN-induced delivery might have specifically targeted the SCS, preventing the potential loss of the drug from diffusing to other areas of the eye. These results suggest that the optimized candlelit-shaped SI-HFMN may serve as a technology for SCS induction and drug delivery to target the PSE. However, because diffusion in the SCS differs depending on the molecular weight, size, and composition of the drugs, further verification using more diverse drugs is needed to confirm the viability of SI-HFMN for SCS delivery [9]. Additionally, further intensive studies are required to fully understand the pharmacokinetics of the drug and its specific distribution within the SCS, especially *in vivo*, which are essential aspects to be considered when translating this technology towards more clinical applications.

## 4 Conclusion

In summary, we developed an optimized SI-HFMN as an innovative drug delivery system to induce SCS formation via swelling and deliver drugs to the PSE. The candlelit-shaped SI-HFMN possessed the maximum swelling volume and area at the depth of the SCS, allowing efficient induction of its formation. The SI-HFMN was able to absorb drugs and release them in a burst release manner, thereby reducing application time. We also demonstrated the ability of the SI-HFMN to penetrate the sclera and swell in a candlelit-shape in *ex vivo* porcine eyes, subsequently inducing the formation of SCS and delivering the absorbed drugs to the PSE. Further studies are needed to verify whether SI-HFMN could deliver drugs to PSE via *in vivo* SCS route in animal models, as well as to evaluate drug efficacy, pharmacokinetics, and safety of the system. Nevertheless, we believe that the SI-HFMN offers a promising platform for minimally invasive and efficient drug delivery to the PSE and addresses pressing contemporary healthcare needs.

## CRediT authorship contribution statement

**Jaibyung Choi:** Writing – original draft, Visualization, Validation, Methodology, Investigation, Formal analysis, Data curation, Conceptualization. **Suhyeon Shim:** Visualization, Validation, Methodology. **Jiwoo Shin:** Writing – review & editing, Writing – original draft, Visualization, Conceptualization. **Ahhyun Lee:** Methodology, Writing – review & editing. **Jaan Strang:** Resources, Funding acquisition, Conceptualization. **Tobias Braun:** Resources, Methodology, Conceptualization. **Reto Naef:** Supervision, Project administration, Funding acquisition, Conceptualization. **Hyungil Jung:** Supervision, Project administration, Funding acquisition, Conceptualization.

## Ethics approval and consent to participate

The manuscript does not involve experimentation on animals and does not include human subjects.

## Declaration of competing interest

Hyungil Jung is an inventor of patents that have been or may be licensed to JUVIC Inc. and is a founder and shareholder of JUVIC Inc., developing microneedle-based products. Reto Naef is a founder of TOPADUR AG. Jaan Strang and Tobias Braun are employees of TOPADUR AG. These potential conflicts of interest have been disclosed by Yonsei University.
