# Supplementary material for: Suprachoroidal space-inducing hydrogel-forming microneedles (SI-HFMN): An innovative platform for drug delivery to the posterior segment of the eye
Source: Bioact Mater. 2025 Apr 3;50:47–60. doi: 10.1016/j.bioactmat.2025.03.024 (PMC11998108; doi:10.1016/j.bioactmat.2025.03.024)
Supplement: Multimedia component 1 [file mmc1.docx]

**Suprachoroidal space-inducing hydrogel-forming microneedles (SI-HFMN): an innovative platform for drug delivery to the posterior segment of the eye**


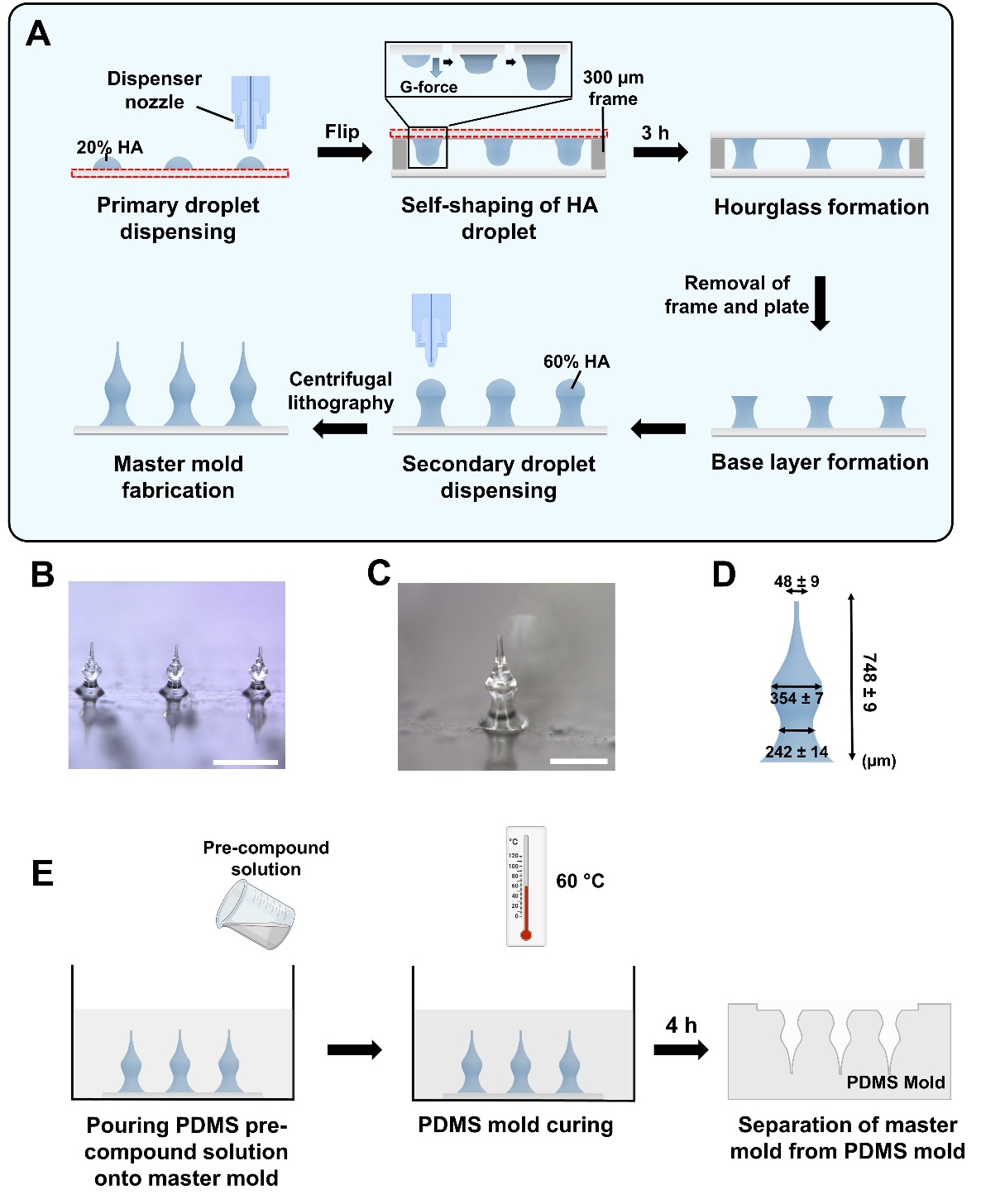


**Fig. S1.** Fabrication process of the candlelit cavity containing polydimethylsiloxane (PDMS) mold. (A) Scheme of candlelit-shaped master mold fabrication process. Fabrication process was divided into two steps, with the first step being the initial base layer formation via g-force and subsequent centrifugal lithography to generate a master mold. Brightfield microscopic images of (B) full 3 × 1 array and (C) single microneedle (MN) (scale bar: 1 mm). (D) Geometric specifications of each MN (n = 9, mean ± SEM). (E) Formation process of PDMS mold from pre-compound solution and prepared master mold.


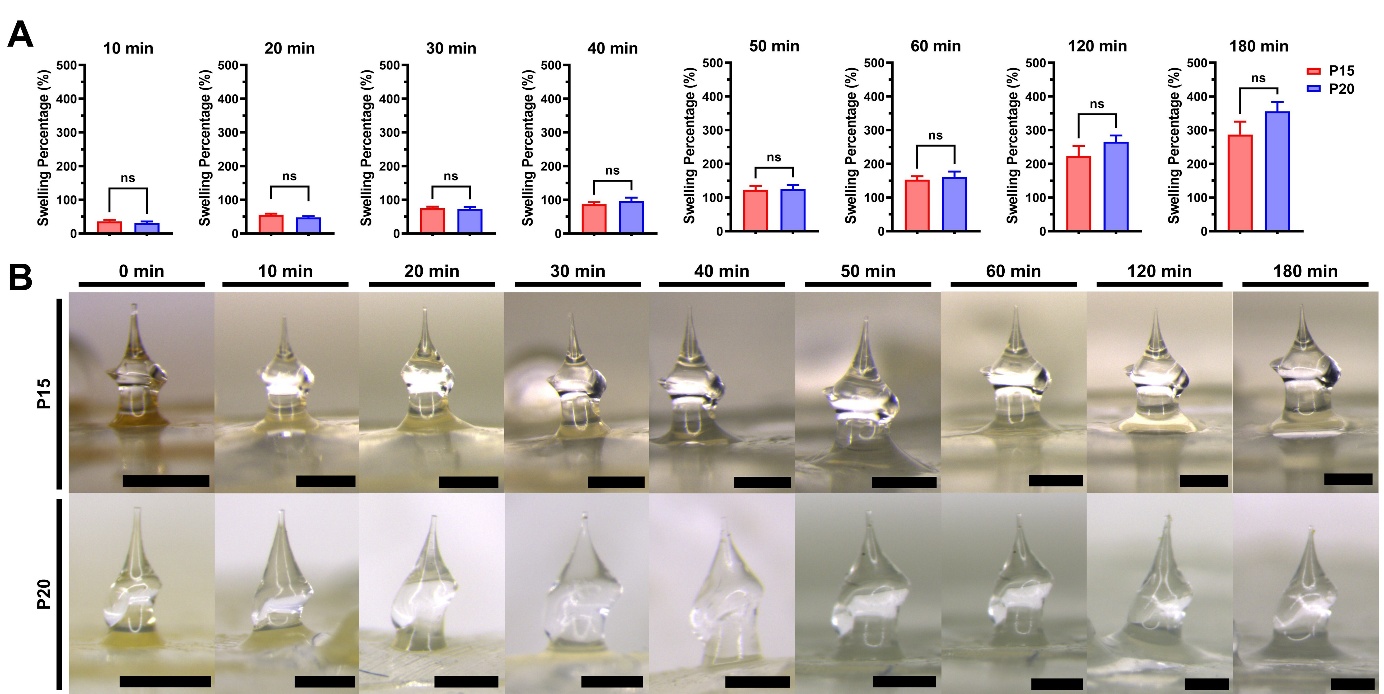


**Fig. S2.** Additional analysis of suprachoroidal space-inducing hydrogel-forming microneedles (SI-HFMN) swelling behavior. (A) Bar graph of *S%* vs. *t* at each time point for each formulation (n = 6, mean ± SEM). ns means not significant. (B) Microscopic images of each SI-HFMN formulation at predetermined timepoints (Scale bar: 500 µm).


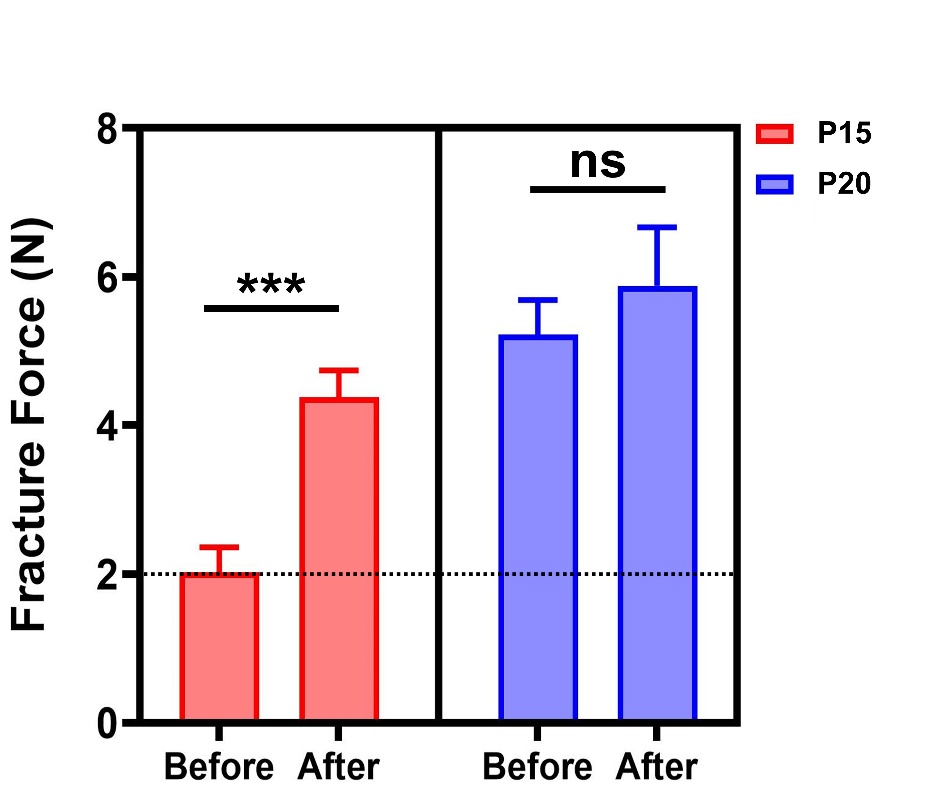


**Fig. S3.** Fracture force evaluation of each suprachoroidal space-inducing hydrogel-forming microneedle (SI-HFMN) formulation after swell/deswell method. Dotted line represents 2.07 N, the minimal force required to penetrate the sclera. All data are presented as mean ± SEM (n = 5). ***, P < 0.001, ns means not significant.


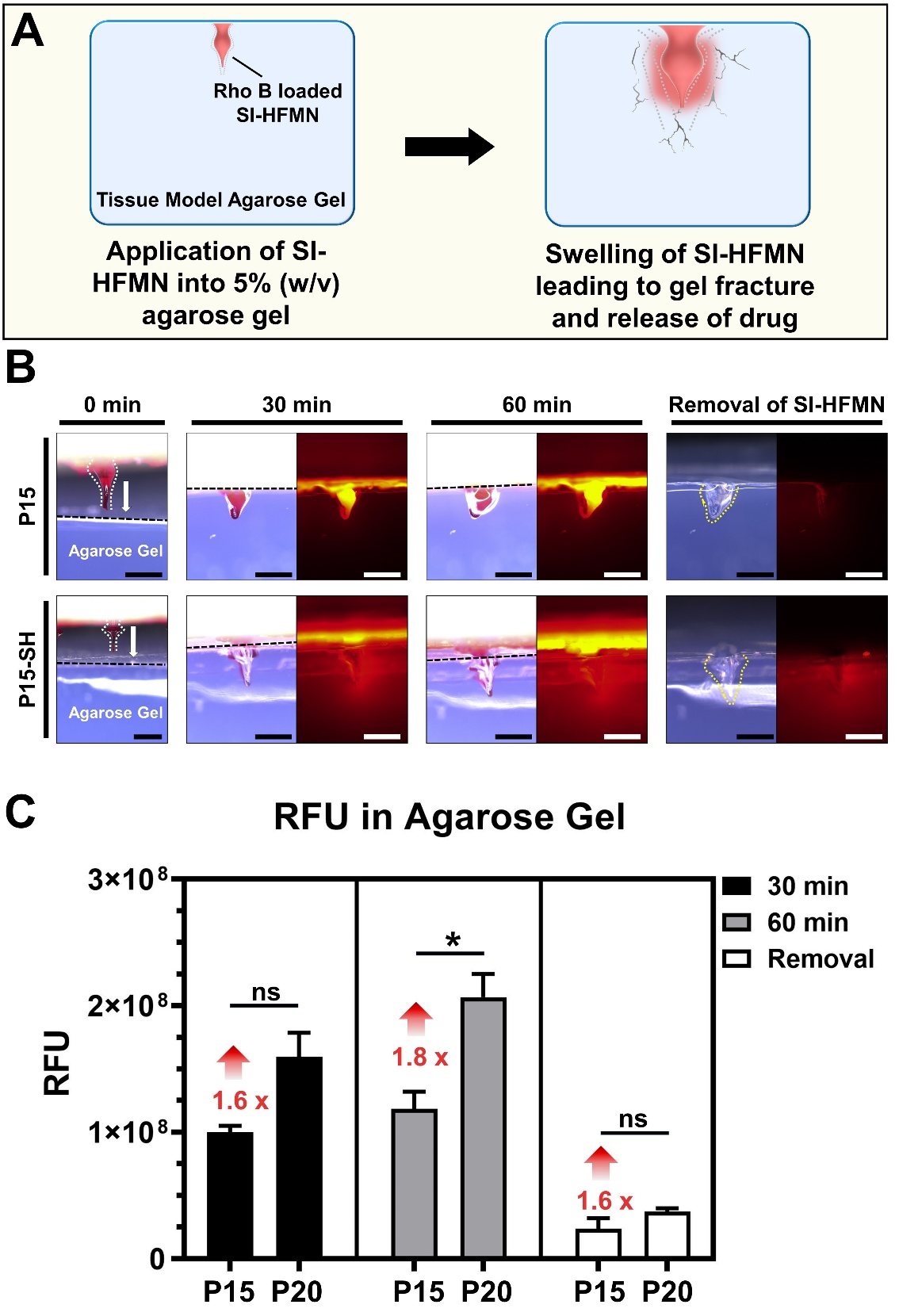


**Fig. S4.** Evaluation of the swelling effect of suprachoroidal space-inducing hydrogel-forming microneedles (SI-HFMN) on *in vitro* tissue model. (A) Overall process of evaluating the swelling effect of SI-HFMN on 5% (w/v) agarose gel. The swelling of SI-HFMN was proposed to both possess sufficient mechanical strength to fracture the stiff gel and release its absorbed drug. (B) Brightfield and fluorescent microscopic images of (top) P15 and (bottom) P20 application on agarose gel at predetermined time points. White arrows show the direction of the SI-HFMN application. Black dotted lines show the application surface of the agarose gel. White dotted lines aid visualization of the Rho B loaded SI-HFMN. The yellow dotted lines indicate area of fracture of agarose gel induced by swelling of SI-HFMN (scale bar: 500 µm). (C) Relative fluorescence intensity unit (RFU) of agarose gel at (black) 30 min, (gray) 60 min, and (white) removal of SI-HFMN for each formulation. Data are presented as mean ± SEM (n = 4). *; P < 0.05, ns means not significant.


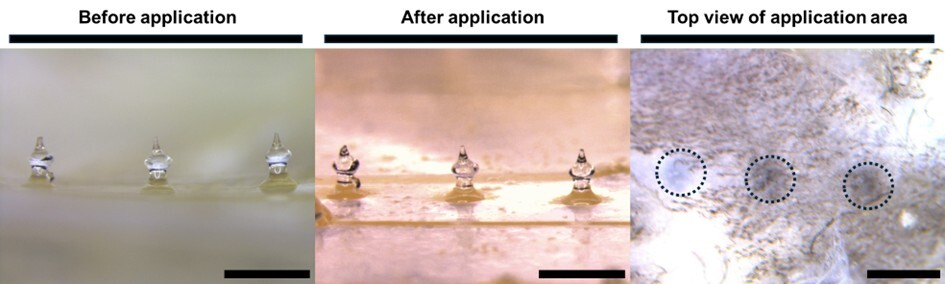


**Fig. S5.** Brightfield microscopic images of blank suprachoroidal space-inducing hydrogel-forming microneedles (SI-HFMN) before (left) and after (middle) application on (right) *ex vivo* porcine cadaver eye (scale bar: 1 mm). Black dotted circles indicate application area.

**Table S1.** Summary of each suprachoroidal space-inducing hydrogel-forming microneedle (SI-HFMN) formulation.

| Formulation | PMVE/MA | PEG | Na_2_CO_3_ |
| --- | --- | --- | --- |
| P15 | 15% | 7.5% | - |
| P20 | 20% | 7.5% | - |
| PNa | 20% | 7.5% | 3% |

**Table S2.** Absorbance Units (AU) value of each formulation at respective peaks of COOH and CO_ester_.

| Absorbance Units | P15 | P20 | PNa |
| --- | --- | --- | --- |
| COOH | 0.01995 | 0.03767 | 0.01855 |
| CO_ester_ | 0.01571 | 0.02027 | 0.00495 |

**Table S3**. Equilibrium water content (EWC) and gel fraction (GF) for each formulation in deionized water (DW)

| Formulation | EWC_DW_ [%] | GF_DW_ [%] |
| --- | --- | --- |
| P15 | 170 ± 1 | 98 ± 1 |
| P20 | 193 ± 2 | 94 ± 1 |

**Table S4.** Nile red absorption parameters of suprachoroidal space-inducing hydrogel-forming microneedles (SI-HFMN)

| Formulation | P*  [cm/s] | K_d_ | D*  [cm^2^/s] |
| --- | --- | --- | --- |
| P15 | 1.6 ± 0.1 | 0.42 ± 0.01 | 2.7 |
| P20 | 1.7 ± 0.1 | 0.42 ± 0.01 | 4.0 |
| * = 10^-5^ | | | |

**Table S5.** Relative Fluorescence Unit (RFU) value of each formulation at various time points.

| Formulation | 30 min* | 60 min* | Removal** |
| --- | --- | --- | --- |
| P15 | 1.0 ± 0.5 | 1.2 ± 0.1 | 2.3 ± 0.9 |
| P20 | 1.6 ± 0.2 | 2.1 ± 0.2 | 3.7 ± 0.3 |
| * = 10^8^, ** = 10^7^ | | | |
